# Contextual-relationship and stress-related factors of postpartum depression symptoms in nulliparas: a prospective study from Ljubljana, Slovenia

**DOI:** 10.1186/s12978-019-0810-x

**Published:** 2019-09-18

**Authors:** Polona Rus Prelog, Marijana Vidmar Šimic, Tanja Premru Sršen, Maja Rus Makovec

**Affiliations:** 1 University Psychiatric Clinic Ljubljana, Studenec 48, 1000 Ljubljana, Slovenia; 2 Division of Gynaecology and Obstetrics, University Medical Centre Ljubljana, Zaloška cesta 7, 1000 Ljubljana, Slovenia; 3 Faculty of Medicine, University of Ljubljana, Vrazov trg 2, 1000 Ljubljana, Slovenia

**Keywords:** Nulliparas, Postpartum depression, Anxiety, Education, Partner attachment

## Abstract

**Background:**

For a significant proportion of women, postpartum depression (PPD) is the first mood episode in their lives, yet its aetiology still remains unclear. Insecure attachment in close adult relationships is considered to be a risk factor for depressive symptoms. This study aimed to gain further insight into the risk factors for postpartum depression symptoms (PPDS) of nulliparas in Slovenia and to examine vulnerability to developing depressive symptoms, with an emphasis on contextual and stress-related characteristics.

**Methods:**

The sample consisted of 156 nulliparas in the third trimester of pregnancy enrolled in a childbirth preparation program. The following instruments were applied: Experiences in Close Relationships-Revised, the Edinburgh Postpartum Depression Scale (EPDS), the Zung Anxiety Scale and a question battery designed by the research team including questions about emotional support and work-related stress. Logistic regression was used to test the association between demographic, social, environmental, personality and attachment variables and PPD of nulliparas (EPDS ≥10), controlling for baseline (prepartum) depression score. A multivariable linear regression model was built with the postpartum EPDS continuous score as a dependent variable**.**

**Results:**

28/156 (17,9%) were evaluated as being at risk for depression (EPDS≥10) in the last trimester and 25/156 (16%) at six weeks postpartum. The results of the logistic regression model controlled for prepartum depression score showed that increased risk for developing PPDS was associated with anxiety level postpartum, intimate-partner-attachment anxiety postpartum, and elevated stress due to loss of employment or an unsuccessful search for employment in the previous year. The results of the multivariable regression model, however, showed the association with education and postpartum anxiety with PPDS continuous score; EPDS after giving birth was higher for more educated and more anxious primiparas.

**Conclusions:**

Our findings demonstrate the importance of anxiety symptoms and higher education level in assessments of nulliparas’ mental health. The results of our study show and confirm the results of previous research that anxiety symptoms in the immediate postpartum period are likely to be associated with depressive symptoms in nulliparas. The results also suggest that higher level of education of first-time mothers might not be a protective factor, especially for nulliparas with the university level of education. Further studies on larger samples should be considered.

## Plain English summary

Depression in women after childbirth (postpartum depression - PPD) is a well-known health-care problem and a serious economic burden. However, it is still often diagnosed late although half of mood disorders actually begin before delivery, in the last trimester of pregnancy.

In recent years, several risk factors have been identified. Those with the strongest impact are previous depressive episodes, previous PPD, anxiety and depressive disorders during pregnancy, which can be predisposed by life stressors and a lack of social support. An intimate relationship and partner attachment style are also considered important. Pregnancy and transition to motherhood can generate stress which can cause insecurely attached women to be more vulnerable to the development of depression.

In this study we further explored the risk factors for depression symptoms of first-time pregnant women. The sample consisted of 156 women in the third trimester in a childbirth preparation program. They answered a questionnaire when 30 weeks pregnant and six weeks after delivery. We found that 17,9% were at risk for depression before and 16% at six weeks after delivery. Increased risk was associated with postpartum anxiety symptoms and higher education of nulliparas. Intimate-partner-attachment anxiety postpartum, and elevated stress due to loss of employment or an unsuccessful search for employment in the previous year were also found important but were not confirmed in the final statistical model. Therefore, we suggest paying attention to anxiety symptoms of first-time mothers. Our results also suggest that higher education of first-time mothers might not be a protective factor for postpartum depression.

## Background

Depression in the postpartum period is a well-known health-care problem and a serious economic burden. For a significant proportion of women, postpartum depression (PPD) is the first mood episode in their lives [1], yet it still often goes unrecognized or is diagnosed late [2, 3]. It has been established that 50% of postpartum mood disorders actually begin prior to delivery and some studies have even shown a higher prevalence of antepartum depression than PPD [4, 5].

Among the factors found to have the strongest impact on the development of PPD are previous depressive episodes, previous PPD, anxiety and depressive disorders during pregnancy [6, 7]. Other predictive factors include a negative birth experience, the mode of delivery (especially Caesarian section), unintended/unplanned pregnancy, lower age and less social support during pregnancy [8, 9].

In recent years, several social, environmental, and stress-related factors have been shown to be associated with PPD. Life stressors and a lack of social support can predispose nulliparas’ anxiety and depressive symptomatology. Support from a partner in pregnancy has been shown to have a predictive value for maternal prenatal and postpartum mental health [10] and an intimate relationship and partner attachment style represent an important part of the emotional experience of nulliparas [11].

Insecure attachment in close adult relationships is considered to be a risk factor for depressive symptoms [12]. Studies have found a prospective association between adult attachment and PPD [13] and some have reported attachment anxiety [14] or insecure attachment without specification of subtype or several subtypes [15–17]. An insecure attachment style in adults is thought to be based on less optimal experiences with early caregiving [18, 19], in addition to many other multifactorial influences [20]. Pregnancy and the transition to motherhood can generate stress and specific concerns with a close relationship, which can cause insecurely attached women to be more vulnerable to the development of depression.

Relatively few studies have analysed the working environment and reported work-related distress as risk factors for PPD [21] when considering environmental factors for a predisposition to depression. Gjerdingen [22] found mothers’ employment to be consistently associated with lower odds of depressive symptoms at 13 months after delivery. A supportive environment and feeling appreciated at work have been found to be important factors in working throughout pregnancy and the working environment might have a favourable effect on women’s health resources [21].

A recent study has found that employment, especially full-time employment and holding a professional or technical job, may reduce the risk of PPD [23]. Work-related and career questions can represent life stress, especially for women with higher education. In the European Union (EU), the proportion of women aged 30–34 with tertiary education exceeds that of men. Most EU countries have a negative gender gap (defined as the proportion of men aged 30–34 with tertiary education minus that of women), with Slovenia showing among the largest gender gaps in tertiary education attainment (− 21.7 p.p. in Slovenia, compared to − 26.0 p.p. in Latvia, which is the largest gender gap in absolute value). In general, women’s age at childbirth has been increasing and their rising educational levels feature prominently among the explanations for increasingly later patterns of age at first birth [24].

While it seems to have been established that PPD is most frequent among women with a history of depression, several prospective studies have shown that a majority of women with depression onset in the postpartum period have no prior history of mood disorders [1, 25]. In fact, a recent systematic review has reported a similar prevalence rate of PPD among healthy mothers without a history of depression when compared to mothers with a history of depression.

Parity has also been found important. Studies have shown higher risk of PPD in nulliparas compared to multiparas, especially in the first month postpartum, and higher scores on the EPDS [26]. A recent study also reported higher fear of childbirth in nulliparas, compared to multiparas [27].

To our knowledge, no studies have so far examined factors associated with PPD in nulliparas in Slovenia. The estimated incidence of postpartum depression in Slovenia is 21% [28]. The population structure of nulliparas in Slovenia has been changing in the last years and decades, the age at first birth is rising, as well as the educational level of women at first birth and they also live in multigenerational households longer (SURS). Identifying risk factors for postpartum depressive symptoms in first-time mothers in this population is therefore highly important. The authors of the recent Cochrane review reported that early identification of mothers at high risk for PPD could help to prevent approximately one-third of the cases of PPD [29]. PPD is difficult to predict and, as several authors have noted, evidence on where to focus screening is still lacking [30, 31].

This study aimed to gain further insight into the risk factors for postpartum depression symptoms (PPDS) of first-time pregnant women (nulliparas) and to examine vulnerability to developing depressive symptoms. Beyond the well-researched risk factors (previous psychiatric illness, history of depression, low income, lower education, poor marital relationship, abuse, previous loss of a baby, etc.), we focused our observation on the contextual and stress-related characteristics of nulliparas.

## Methods

### Study sample

The sample of the present study consisted of 156 pregnant women in the third trimester recruited sequentially from parenting classes held at the University Medical Centre (UMC) Ljubljana’s Division of Gynaecology and Obstetrics from March to September 2014. The inclusion criteria were: first pregnancy, third trimester of pregnancy, at least 18 years of age. Excluded from the study were the women who did not fill out the whole EPDS questionnaire at both assessment points.

At baseline, there were 325 women included in the study, of which 181 (55,7%) at least partially completed the questionnaires before and after giving birth. Of these, 156 had complete data on the dependent variable (EPDS score). These were included in the statistical analysis.

Women that were included in the statistical analysis (*n* = 156) did not differ from women initially included in the study in any of the variables but in positive experience of birth (*p* = 0,002) and baseline attachment anxiety (p = 0,012). Women with positive experience of birth and lower baseline attachment anxiety were less likely to drop out of the study.

From 156 women with complete data on EPDS, 90 (57,7%) women were included in the multivariable linear regression model due to missing data and listwise deletion.

A statistically significant difference between missing and non-missing cases was found in the variable measuring stress due to primiparas’ physical or mental illness. Women experiencing high stress due to their own physical or mental illness in the last 12 months were less likely to provide complete answers (*p* = 0.013). There were no statistically significant differences between women included and excluded from the regression model analysis in any other categorical or continuous variable. The demographic characteristics of the sample are listed in Table 1.

**Table 1:** Characteristics of primiparas (results shown as frequency and percentages if not indicated differently)

	n = 156
Mean age (SD)	30.7 (4.1)
Mean years of education (SD)	16.4 (2.3)
Planned pregnancy	129 (82.7)
Caesarean section (*n* = 150)	32 (21.3)
Positive birth experience (*n* = 152)	126 (82.9)
Multigenerational household (*n* = 155)	33 (21.3)
Constant support from partner (n = 150)	131 (87.3)
Constant support from parents (n = 150)	94 (62.7)
Constant support from friends (*n* = 151)	66 (43.7)
High support from co-workers b.b.^a^(*n* = 147)	66 (44.9)
Mean attachment avoidance bb (SD) (*n* = 144)	1.8 (0.7)
Mean attachment anxiety bb (SD) (*n* = 141)	1.7 (0.6)
Mean attachment avoidance ab (SD) (*n* = 135)	1.8 (0.8)
Mean attachment anxiety ab (SD) (n = 144)	1.7 (0.7)
Stress - loss of employment (n = 150)	15 (10)
Stress – family member or close friend’s illness or injury (n = 151)	27 (17.9)
Stress - own illness or injury (*n* = 149)	10 (6.7)
Stress - financial difficulties (n = 150)	19 (12.7)
Psychiatric help before pregnancy (n = 155)	16 (10.3)
Mean anxiety score bb (SD) (n = 144)	33.2 (5.7)
Mean anxiety score ab (SD) (n = 150)	29.6 (5.8)

^a^*bb* before giving birth, *ab* after giving birth

### Procedure

The study was conducted as a collaboration between obstetricians from UMC Ljubljana’s Division of Gynaecology and Obstetrics and psychiatrists from the University Psychiatric Hospital Ljubljana. It was approved by the Republic of Slovenia National Medical Ethics Committee (NMEC) (protocol No. 92/12/13). All of the study participants were given verbal and written explanations of the study and their informed consent was obtained prior to their participation in the study. The study was based on a convenience sample.

The questionnaires were administered during parenting classes from March 2014 to September 2014. The classes are run by midwives and include lectures by a paediatrician, an anaesthesiologist, a dentist, psychologists, and other specialists. The classes are open to pregnant women in their third trimester and are mostly attended by women in their first pregnancy; their partners are welcome to attend. Each class consists of 10 meetings over three weeks. The topics are preparation for labour, birth, and postnatal care of the baby.

The participants completed a structured questionnaire in their third trimester of pregnancy (mean 30 weeks). Each participant was given an anonymous questionnaire with a code, which was saved together with their personal information. During the recruitment period, the midwives and participating doctors invited 696 Slovenian-speaking Caucasian pregnant women who attended the classes to take part. Written informed consent to participate was signed by 387 (55.6%) of the women. Participants completed the second questionnaire six weeks after giving birth via the online tool SurveyMonkey. Participants who did not answer the follow up received a maximum of two e-mail reminders. We excluded 38 of the participating women who were in their second or subsequent pregnancies.

A prospective longitudinal design was used. We used SRQR reporting guidelines [32].

### Measures/instruments

#### Partner attachment

Experiences in Close Relationships-Revised (ECR-R) [33]: the ECR-R is a 36-item self-report measure used to assess adult romantic attachment on a 7-point scale ranging from 1 (strongly disagree) to 7 (strongly agree). The scale consists of two 18-item subscales: anxiety (fear of rejection and abandonment) and avoidance (discomfort with closeness and discomfort with depending on others). For our sample, internal consistency was α = .84 both for the Avoidance and the Anxiety scale.

#### Depressive symptoms

The Edinburgh Postpartum Depression Scale (EPDS) [34]: The EPDS is a self-report questionnaire consisting of 10 items with four ordered response categories scored from 0 to 3. When used as a screening instrument, the cut-off scores of 12/13 usually designate major depression, whereas scores from 9 to 11 indicate mild depression levels in need of further assessment [35]; Cronbach α = .83. We used a cut-off score = 10, considering that this cut-off proved reasonable/appropriate in a previous study using a Slovene sample [36].

#### Anxiety

The Zung Anxiety Scale [37] consists of 20 items which test the participants’ autonomic, motor, cognitive and other anxiety symptoms. For each item the participants choose one of the following answers: a little of the time, some of the time, good part of the time, most of the time; Cronbach α = .76.

#### Sociodemographic and pregnancy information

A question battery designed by the research team included: maternal age; years of education (education level); planned/unplanned pregnancy; living arrangement (shared household with elder generation/own household); mode of delivery (Caesarian section); whether psychiatric help had been received before pregnancy; level of emotional support received from partner, parents, friends and co-workers; stress due to loss of employment/illness/financial problems.

Constant (before and after giving birth) emotional support from partner, parents, friends and emotional support from co-workers during pregnancy was measured on a 5- point self-constructed scale containing 4 questions. The scale that was dichotomized prior to analysis into categories “weak to moderate support” including answers “almost none” to “moderate support” and “strong support” including answers “strong” and “very strong support”.

Stress in the last year due to unemployment, family member or close friend’s illness, nulliparas’ physical or mental illness and financial problems was measured on a 5-point scale that was dichotomised prior to analysis (1 thru 3 = low stress; 4 thru 5 = high stress).

### Statistical analysis

Logistic regression was used to test the association between demographic, social, environmental, personality and attachment variables and PPD of nulliparas, controlling for prepartum depression score. PPD was indicated by an EPDS score higher or equal to 10 points. As there was a smaller number of nulliparas with afterbirth depression score above or equal to 10 points (*n* = 25), no multivariable logistic regression model was built, since no fewer than 10 cases in the smaller category of the dependent variable per included independent variable is recommended to obtain valid and stable results of the logistic regression model [38, 39]. In the current study up to 2 independent variables could be included in the logistic regression model.

To assess which risk factors play the most important role in postpartum depression, a multivariable linear regression model was built with the continuous after-birth EPDS score as a dependent variable.

The independent variables in each model were: age; years of education; planned pregnancy (yes/no); Caesarean section (yes/no); positive birth experience (yes/no); shared household with elder generation (yes/no); constant (before and after giving birth) emotional support from a partner, parents and friends and emotional support from co-workers during pregnancy (weak to moderate = almost none to moderate support; strong = strong and very strong support); attachment avoidance and attachment anxiety before and after giving birth; stress in the last year due to unemployment or illness of a family member or close friend; nulliparas’ physical or mental illness; financial problems (1 through 3 = low stress; 4 through 5 = high stress); psychiatric help before pregnancy (yes/no); Zung’s anxiety score before and after giving birth and the EPDS before giving birth. Each of the above-mentioned risk factors was included in the logistic model, controlled for prepartum depression score of nulliparas. Adjusted odds ratios for being at risk for postpartum depression (postpartum EPDS score ≥ 10) were calculated.

The same risk factors were then included in the multivariable linear regression model, where the dependent variable was EPDS postpartum score (continuous variable). There was no threat of multicollinearity as the highest variance inflation factor was 2.9.

*P*-values < 0.05 (two-tailed) were treated as statistically significant. No adjustment for multiple comparisons was applied. Data were analysed using SPSS version 24 for Windows.

## Results

### Prevalence of PPD in the study sample

One hundred fifty-six nulliparous women who answered the questionnaire in the last trimester and six weeks postpartum were included in the study. Of those, 28/156 (17,9%) were evaluated as being at risk for depression (EPDS≥10) in the last trimester and 25/156 (16%) at six weeks postpartum.

### Socio-demographic characteristics of study subjects

The characteristics of primiparas that completed the questionnaire before and after giving birth are summarized in Table 1.

### Results of logistic regression and multivariable linear regression analysis

The results of the logistic regression showed that, when controlled for the prepartum depression score, EPDS after giving birth above or equal to 10 points was associated with higher anxiety score and higher attachment anxiety postpartum and with stress due to loss of employment (Table 2). Other risk factors were not associated with being at risk for PPD (EPDS score ≥ 10).

**Table 2:** Association between demographic, social and environmental factors and a high EPDS (results of adjusted logistic regression)

	D.score < 10	D.score > =10	aOR^a^ (95%CI)	P
Mean age (SD; n)	30.6 (4.1; 131)	31.1 (4; 25)	1 (0.9; 1.2)	0.442
Mean years of education (SD; n)	16.4 (2.3; 131)	16.5 (1.8; 25)	1 (0.8; 1.2)	0.888
Planned pregnancy	109/131 (83.2)	20/25 (80)	0.9 (0.3; 2.9)	0.913
Caesarean section	25/126 (19.8)	7/24 (29.2)	1.8 (0.7; 4.9)	0.251
Positive birth experience	108/130 (83.1)	18/22 (81.8)	0.9 (0.3; 3.1)	0.685
Multigenerational household	26/130 (20)	7/25 (28)	1.4 (0.5; 3.8)	0.517
Constant support from partner	110/125 (88)	21/25 (84)	0.9 (0.3; 3.5)	0.982
Constant support from parents	78/125 (62.4)	16/25 (64)	1.1 (0.5; 2.8)	0.798
Constant support from friends	57/126 (45.2)	9/25 (36)	0.8 (0.3; 1.9)	0.554
High support from co-workers bb	60/124 (48.4)	6/23 (26.1)	0.4 (0.1; 1.1)	0.087
Mean attachment avoidance bb (SD; n)	1.8 (0.7; 121)	2.1 (0.7; 23)	1 (1; 1.1)	0.176
Mean attachment anxiety bb (SD; n)	1.6 (0.5; 119)	2 (0.9; 22)	1.04 (1; 1.08)	0.075
Mean attachment avoidance ab (SD; n)	1.8 (0.7; 114)	2.1 (0.8; 21)	1 (1; 1.1)	0.217
Mean attachment anxiety ab (SD; n)	1.6 (0.6; 122)	2.3 (1.1; 22)	1.1 (1; 1.1)	0.005
Stress – loss of employment	8/125 (6.4)	7/25 (28)	5.5 (1.7; 17.3)	0.004
Stress – family member or close friend’s illness or injury	21/126 (16.7)	6/25 (24)	1.4 (0.5; 4.1)	0.500
Stress – own illness or injury	7/124 (5.6)	3/25 (12)	2 (0.5; 8.8)	0.335
Stress – financial difficulties	15/126 (11.9)	4/24 (16.7)	1 (0.3; 3.7)	0.983
Psychiatric help before pregnancy	14/130 (10.8)	2/25 (8)	0.8 (0.2; 4)	0.826
Mean anxiety score bb (SD; n)	32.8 (5.5; 121)	35.6 (6; 23)	1.1 (1; 1.2)	0.109
Mean anxiety score ab (SD; n)	28.3 (4.8; 126)	36.3 (5.7; 24)	1.3 (1.2; 1.5)	< 0.001

^a^*OR* odds ratio, *D. score* Edinburgh depression score, *aOR* OR adjusted for baseline depression score

There was a moderate correlation between Zung’s anxiety score before and after giving birth (r = 0.44; *p* < 0.001). The anxiety score after giving birth on average decreased by 4 points (SD = 6; p < 0.001).

There was a strong correlation between attachment avoidance before and after giving birth (0.71; p < 0.001), and between attachment anxiety before and after giving birth (r = 0.53; p < 0.001). The results suggest that anxiety and anxious attachment style are prone to change after giving birth. This explains the decision of including the before and after birth measures in the regression model separately.

From all the predictors included in the multivariable linear regression model, only education and anxiety after giving birth were positively associated with the depression score after giving birth, when other predictors were controlled for. The EPDS after giving birth was higher in more educated and more anxious nulliparas (Table 3). By the regression model, 60% of variance in the EPDS is explained.

**Table 3:** Association between demographic, social, environmental and personality variables and EPDS (results of multiple regression analysis)

	Std. B^a^ (P)
Age	0.09 (0.348)
Years of education	0.22 (0.029)
Planned pregnancy	−0.04 (0.727)
Caesarean section	0.08 (0.438)
Positive birth experience	0.07 (0.483)
Multigenerational household	0.1 (0.317)
Constant support from partner	−0.15 (0.12)
Constant support from parents	−0.02 (0.88)
Constant support from friends	0.08 (0.482)
High support from co-workers bb	0.09 (0.383)
Attachment avoidance bb	−0.02 (0.907)
Attachment anxiety bb	0.1 (0.482)
Attachment avoidance ab	0.01 (0.964)
Attachment anxiety ab	0.08 (0.561)
Stress – loss of employment	−0.04 (0.654)
Stress – family member or close friend’s illness or injury	0 (0.992)
Stress – own illness or injury	−0.04 (0.682)
Stress – financial difficulties	−0.07 (0.538)
Psychiatric help before pregnancy	−0.03 (0.79)
Anxiety score bb	−0.12 (0.34)
Anxiety score ab	0.72 (< 0.001)
Depression score bb	0.1 (0.448)

^a^*Std. B* standardized regression coefficient

### Summary of results

We explored various factors associated with postpartum depression in first-time pregnant women that could represent increased risk for developing postpartum symptoms of depression in first-time pregnant women.

The results of logistic regression models showed that increased risk for developing PPDS was associated with anxiety postpartum, intimate-partner-attachment anxiety postpartum, and elevated stress due to loss of employment or an unsuccessful search for employment in the previous year, when controlled for the prepartum depression score.

However, the results from the multivariable linear regression model demonstrated that only education and anxiety after birth were associated with postpartum depression in nulliparas when other predictors were controlled for. The EPDS after giving birth was higher for more educated and more anxious nulliparas.

## Discussion

Multivariable regression analysis showed that the perception of women’s experience of childbirth in our sample was not significantly related to higher scores on the EPDS. Of note, women with positive experience of delivery were less likely to drop out of the study. A recent meta-analysis [9] reported that Caesarean sections increased the risk of PPD. However, in our sample, we found no relationship between Caesarean sections and PPD.

The relationship between unplanned pregnancy and postpartum level of depression (higher EPDS score) in our sample was nonsignificant, which is inconsistent with a recent meta-analysis that found the prevalence of perinatal depression to be twice as high in women with an unintended pregnancy [40], but consistent with other longitudinal studies reporting statistically nonsignificant associations between unintended pregnancies and PPD [41, 42].

In contrast with previous studies that reported lack of social support to be a risk factor for PPD [43, 44], we found that the perception of emotional support from a partner, parents, friends or co-workers during pregnancy was not significantly related to higher scores on the EPDS. This finding might also be due to the relatively stable relationships of women who participated in our study. A majority of the participants (87.3%) reported constant support from partner, 62.7% reported constant support from parents, 43,7% reported constant friends’ support and 44,9% reported support from co-workers.

Partner- attachment anxiety postpartum was found to be positively associated with EPDS in the univariate logistic model, which is consistent with the results of other studies; anxious styles were found to be associated with PPDS more frequently than avoidant styles of attachment [13, 14]. The multivariable regression model, however, did not confirm the association of attachment style and PPD.

Elevated stress due to loss of employment or an unsuccessful search for employment in the previous year was associated with higher odds for PPDS in the logistic regression model. In line with the results of our study, a recent study found employment, compared with unemployment, to be significantly associated with a reduced risk for postpartum depression [23]. However, in our study, the association was not confirmed in the multivariable regression model.

Several studies have demonstrated depression and anxiety during pregnancy to be risk factors for PPD [6, 7]. Some studies have even shown anxiety disorders during pregnancy to be a stronger predictor of PPD than depression [45, 46]. In our sample, however, only anxiety postpartum was related to PPDS and the correlation between postpartum anxiety and PPDS was highly significant in both statistical models. One obvious explanation for this could be the overlap of symptoms as well as comorbidity, which is in line with the results of previous research that found anxiety disorders to be highly comorbid with peripartum depression [47]. A recent study reported that 13% of women experienced comorbid depressive symptomatology and anxiety during the first eight weeks postpartum [48, 49]. The more anxious nulliparas in our study also experienced higher levels of depression.

In the current study, the prevalence of being evaluated as being at risk for depression was 17,9% in the third trimester and 16% six weeks after birth. This is in line with, or slightly higher than, what has been reported in previous studies [13, 30], possibly due to the cut-off of EPDS≥10. Similarly, the study on a sample of 449 Slovenian new mothers found that 21.3% of the new mothers had a score of 10 or higher on the EPDS, but the reported study did not exclude multiparas, therefore our results cannot be directly compared [28].

Interestingly, a history of psychiatric help before pregnancy was not related to higher scores on the EPDS. Ten per cent of women in our sample reported getting psychiatric help before pregnancy, yet we found no association with PPD in any of the models, which further emphasizes the importance of searching for less obvious risk factors.

In the current study, higher education was related to higher depression levels, in contrast to studies demonstrating lower education to be associated with PPD [50, 51]. We could speculate that, for highly educated women, taking maternity leave and starting a family represents an additional challenge in their career development and can generate stress that could increase vulnerability to PPDS. Based on this, we might consider the cognitive dissonance of nulliparous women who want an active role in their career. For the highly educated women of our study, pregnancy and the transition to parenthood might have been especially important for their mood level.

### Strengths & Limitations

This study has some limitations. Most of the participating women in our study were highly educated first-time pregnant women living in an urban environment which might not be representative of the Slovenian population and the results should probably not be generalized. Possible reasons for these limitations are the use of a university hospital for the study and the inclusion of Slovenian-speaking women only.

The participation rate in our study was approximately 55%, which is in line with similar studies [30]. Of note, nulliparas with positive experience of birth and lower baseline attachment anxiety were less likely to drop out of the study, while women experiencing high stress due to their own physical or mental illness in the last 12 months were less likely to provide complete answers (*p* = 0.013), which might have led to an underestimation of PPD and factors associated with higher EPDS our sample.

When compared to other nulliparas who gave birth in University Medical Centre (UMC) Ljubljana’s Division of Gynaecology and Obstetrics in the year 2014, the women in our sample were more educated and slightly older (statistical significance is not known). The mean years of education in our sample was 16,4 (higher education–university level), while 37.3% of nulliparas that gave birth in 2014 had 16 years of education (university level). The mean age of nulliparas that gave birth in 2014 was 29.35, compared to 30.7 years in our sample.

Our diagnostic assessment focused on mood symptoms, not disorders.

Finally, the sample size of our study was rather small. One of the reasons might be the length of the questionnaire and time/schedule limitations of first-time mothers 6 weeks after delivery, which might also account for the missing data. Future studies should focus on trying to have larger sample sizes.

The strengths of the study include the prospective design, where stress-related and other factors were measured in the last trimester of pregnancy and six weeks after birth, and the contextual framework/design.

### Implications

Some practical implications may be taken from the present findings. Knowledge of the relative importance of risk factors associated with PPDS of first-time pregnant women might add to the recognition of vulnerable women and also help develop effective clinical interventions. The peripartum period is a time when most women are in routine contact with health professionals (e.g. midwives). Attention to symptoms of anxiety when assessing peripartum mental health might improve earlier recognition of PPDS and enable early preventive or treatment interventions. Sub-threshold symptom recognition is important in this critical time of early postpartum, when non-pharmacological interventions are of even greater importance. Including questions about experiencing anxiety symptoms in peripartum questionnaires might prove useful in screening for high risk of PPDS.

## Conclusions

Our findings demonstrate the importance of anxiety symptoms and higher education level in assessments of nulliparas’ mental health. To our knowledge, our study is unique in exploring the contextual understanding of factors, associated with nulliparas’ PPDS in a group of first-time pregnant women in Slovenia.

The results of our study show and confirm the results of previous research that anxiety symptoms in the immediate postpartum period are likely to be associated with depressive symptoms in nulliparas. The results also suggest that higher level of education of first-time mothers might not be a protective factor, especially for nulliparas with university level of education.

Attention to the symptoms of anxiety in highly educated nulliparas with seemingly protective psychosocial determinants might add to recognition of vulnerable women in assessment of PPDS.

The logistic regression model showed the association of anxious attachment style and unemployment with increased risk of PPD; however, given that the significant findings from the univariate analysis were not confirmed in the multivariable regression model, we suggest further studies with larger samples.

## Data Availability

The data are not publicly available due to their containing information that could compromise research participant privacy/consent. The data are available from the corresponding author on reasonable request.

## Abbreviations

ECR-R: Experiences in Close Relationships-Revised
EPDS: The Edinburgh Postpartum Depression Scale
EU: European Union
NMEC: National Medical Ethics Committee of Republic of Slovenia
PPD: Postpartum depression
PPDS: Postpartum depression symptoms
UMC: University Medical Centre Ljubljana’s Division of Gynaecology and Obstetrics
